# Lethal Pulmonary Embolism Following Left Upper Extremity Angiogram/Angioplasty with Thrombectomy at Malfunctioning Arteriovenous Fistula

**DOI:** 10.7759/cureus.7197

**Published:** 2020-03-06

**Authors:** Kenneth K Ng, Tatyana Rozental

**Affiliations:** 1 Anesthesiology, State University of New York Downstate Medical Center, Brooklyn, USA

**Keywords:** pulmonary embolism, cardiothoracic anesthesia, vascular surgery, arteriovenous fistula repair, thrombectomy, arteriovenous fistula, angiogram, angioplasty, end-stage renal disease (esrd), revascularization

## Abstract

A 41-one-year-old female on chronic hemodialysis with a medical history of systemic lupus erythematosus and end-stage renal disease presented with clotted hemodialysis access. She was sent to the operating room for angiogram/angioplasty with thrombectomy. Although the thrombectomy was successful, toward the end of the case, she went into cardiac arrest secondary to a massive pulmonary embolization that probably originated from her clotted arteriovenous fistula. Spontaneous circulation returned only after a prolonged period of resuscitation using advanced cardiac life support. She was transferred to the medical intensive care unit (ICU) intubated while receiving minimal cardiovascular pharmacological support. Computerized tomography (CT) scan of the head indicated extensive anoxic brain injury and she expired two days later.

## Introduction

End-stage renal disease (ESRD) is a major cause of disease and death in the United States (US). The overall prevalence of chronic kidney disease (CKD) is 14% in the general population and approximately 661,000 Americans have Stage 5 ESRD [1]. Stage 5 ESRD is fatal without hemodialysis or a kidney transplant due to the accumulation of uremic toxins. In the United States, approximately 468,000 patients depend upon hemodialysis for survival and 193,000 have a functioning kidney transplant [1].

Patients on chronic hemodialysis have three main options for vascular access during hemodialysis: fistulas, grafts, and catheters. An arteriovenous fistula (AVF) is generally the preferred choice for access due to greater mechanical strength, lower risk of clotting or infection, and an ability to last longer than other vascular options. Nevertheless, grafts or catheters are options for patients with weaker arteries or veins or those who need only temporary hemodialysis [2].

Chronic kidney disease and Stage 5 ESRD have established risk factors for both arterial and venous thrombophilia with odds ratios of 2.5 and 5.5, respectively, compared to the general population [3]. Stage 5 ESRD patients have been shown to be hypercoagulable with an increased prevalence of thrombophilic factors, including factor VIIIC, lupus anticoagulant, and antiphospholipid antibodies. They also exhibit protein C deficiency, protein S deficiency, and activated protein C resistance [4]. It can be assumed that the risk of thrombophilia is even greater in patients with other risk factors for hypercoagulability, such as this patient with systemic lupus erythematosus (SLE).

Angiogram and angioplasty with thrombectomy of a clotted upper extremity AVF is a commonly performed surgical intervention in ESRD patients on chronic hemodialysis. The objective of angiogram and angioplasty is to determine the patency/functionality of the AVF, mechanically/chemically remove clots blocking the AVF, and restore functionality to the existing fistula, obviating the need for surgical creation of a new AVF which may take one to four months to become active.

This case study demonstrates that this procedure is not without risks in itself, and both surgeons and anesthesiologists should be cognizant of the possibility of major morbidity and mortality resulting from the embolization of clots present in the AVF.

## Case presentation

The patient was a 41-year-old African-American female with a past medical history of SLE and ESRD (likely secondary to lupus nephritis). She had been receiving outpatient hemodialysis three days per week for eight years. The patient presented to the emergency department due to left upper extremity pain and swelling secondary to clotting in her left AVF. She was dialyzed with temporary access placed in her right groin and scheduled for left AVF angiogram, angioplasty, and thrombectomy with a possible stent and banding.

The patient was brought to the operating room the following day. She was given options for either general anesthesia or monitored anesthesia care (MAC). She chose general anesthesia. She was NPO (nothing per mouth) for eight hours before the procedure, given half doses of diabetic medications on the day of the procedure, and continued all antihypertensive medications. Venous blood gas drawn in the preoperative area showed hyperkalemia at 5.8. She was pre-treated with albuterol, intravenous regular insulin, and dextrose, 50 grams, before the procedure. The patient was intubated and induced without issue utilizing midazolam, fentanyl, lidocaine, propofol, and rocuronium. She was maintained on sevoflurane, 2.5%, and periodically given fentanyl for pain control and re-paralyzed with rocuronium. She was given heparin, 4,000 units prophylactically, at the start of the procedure. Her hemodynamics were stable throughout most of the procedure with a mean arterial pressure of 70 - 80 mmHg.

At 2.5 hours intraoperatively, after the angiogram was completed and the surgeon was performing thrombolysis of the clots, the patient suddenly became hypotensive to 40/20 mmHg with a significant decrease observed in end-tidal carbon dioxide (CO_2_) from 35 to 12 mmHg. Her pulse was not palpable and she did not respond to multiple injections of intravenous (IV) phenylephrine or ephedrine. Advanced cardiac life support (ACLS) was activated in the operating room and chest compressions were initiated with the administration of IV epinephrine every 3 - 5 minutes. She was also given IV calcium and bicarbonate when her arterial blood gas showed significant metabolic acidosis. A bedside transthoracic echocardiogram revealed a very dilated, severely hypokinetic right ventricle, indicating a massive pulmonary embolism. Tissue plasminogen activator (tPA) and heparin were administered. A femoral central line was placed and continuous infusions of norepinephrine, vasopressin, and epinephrine were initiated. Return of spontaneous circulation was achieved after a prolonged period of chest compressions and resuscitation.

**Figure 1:**
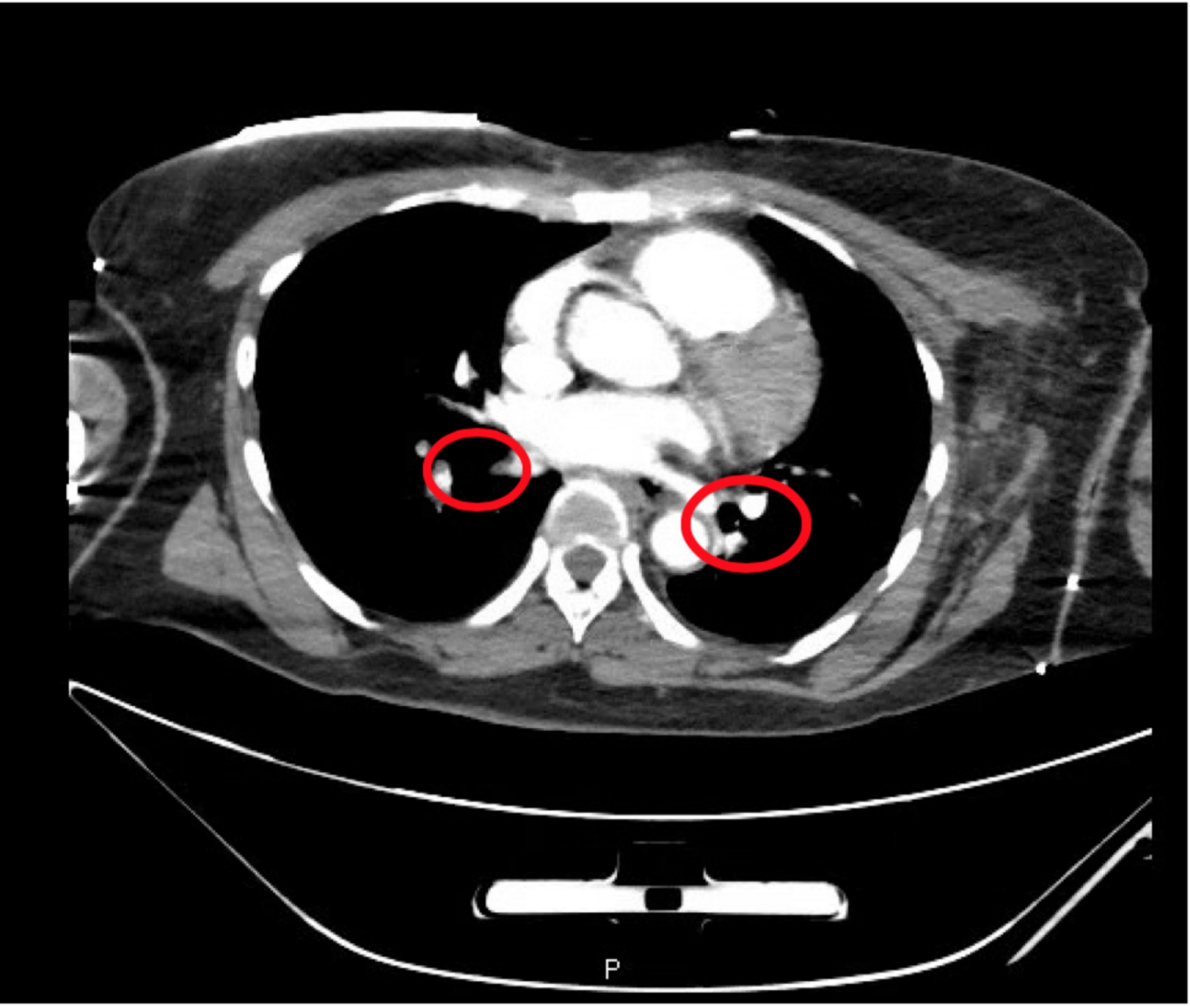
Computed tomography angiogram (CTA) with contrast demonstrates large bilateral subsegmental pulmonary embolisms in the truncus anterior and posterior basal segments

**Figure 2:**
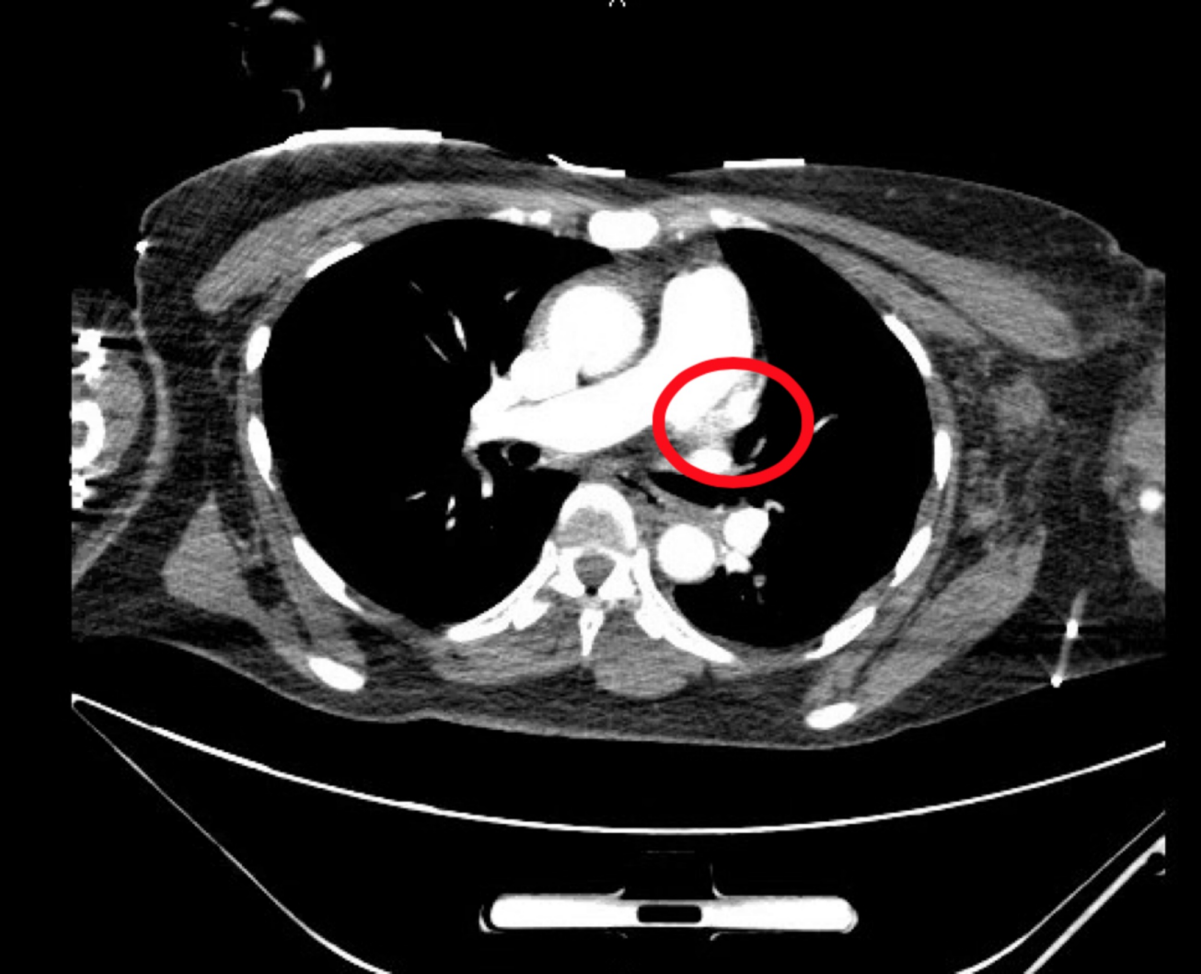
Computed tomography angiogram shows a large filling defect in the main left pulmonary artery consistent with a massive pulmonary embolism. Note also dilation of the pulmonary trunk and right main pulmonary artery.

**Figure 3:**
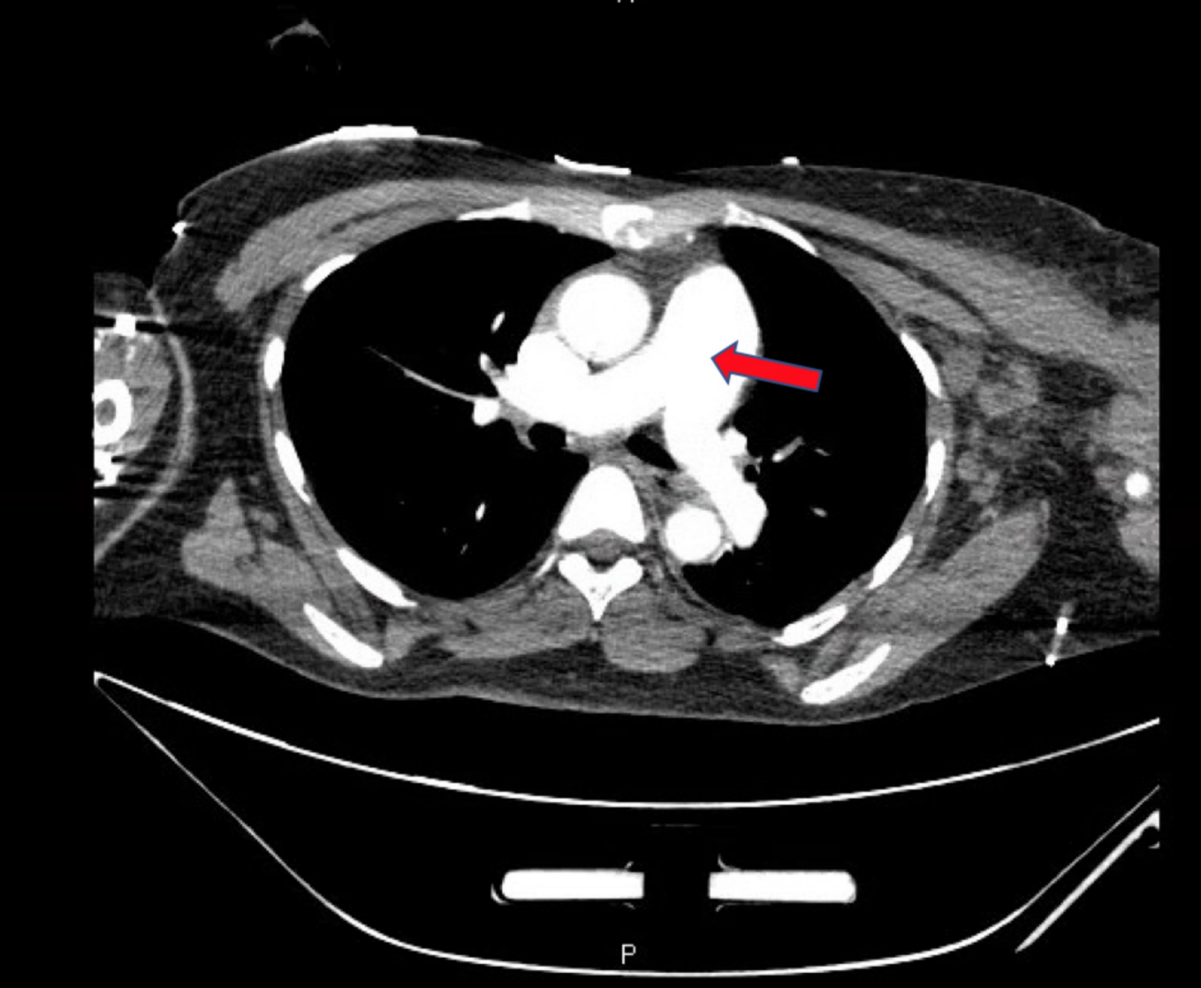
Massive dilation of the main pulmonary artery consistent with a large subsegmental pulmonary emboli and right ventricular heart strain

**Figure 4:**
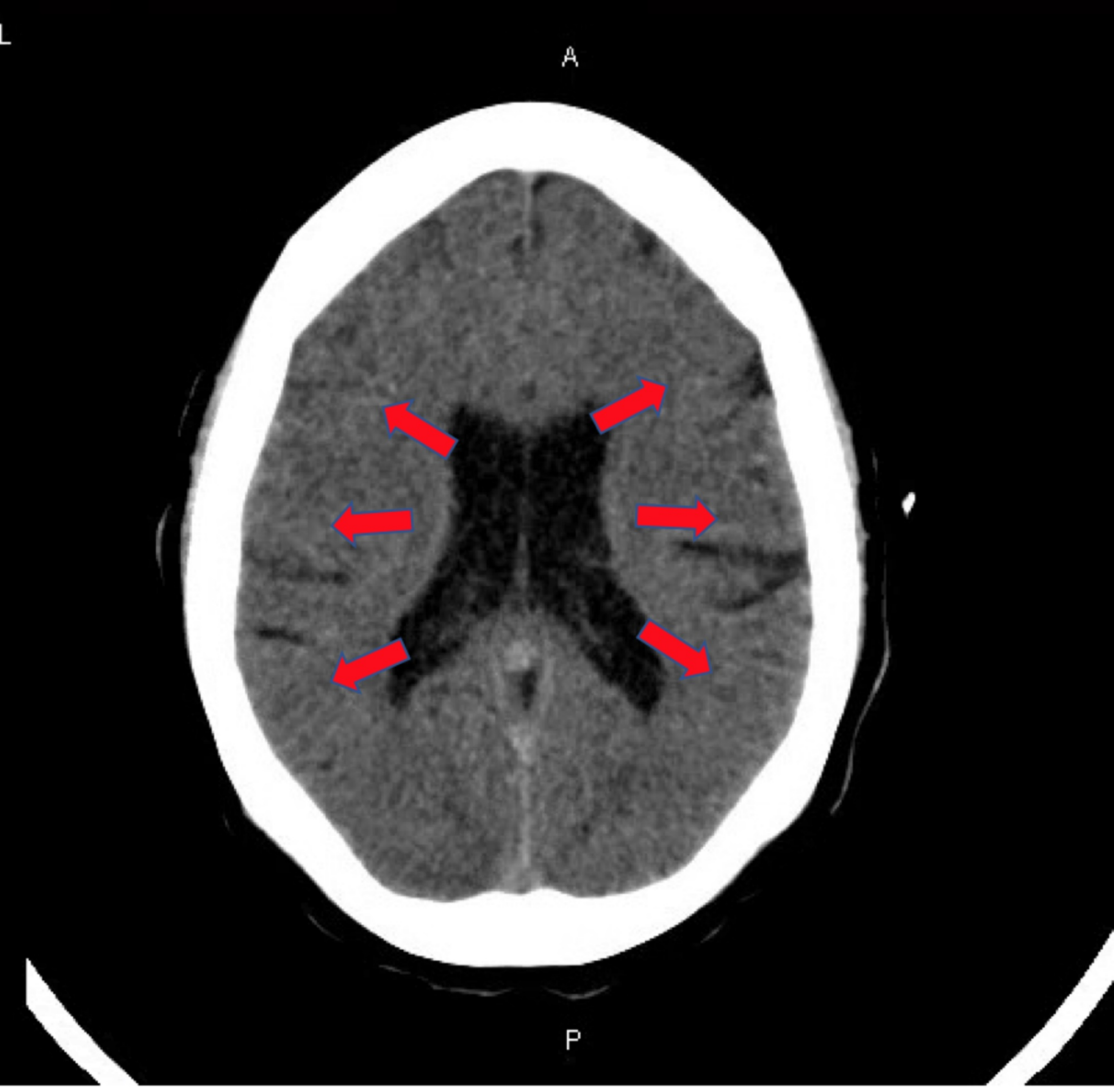
Computed tomography of the head without contrast shows loss of gray-white matter differentiation and diffuse anoxic brain injury consistent with hypoxic ischemic encephalopathy in the setting of cardiac arrest

**Figure 5:**
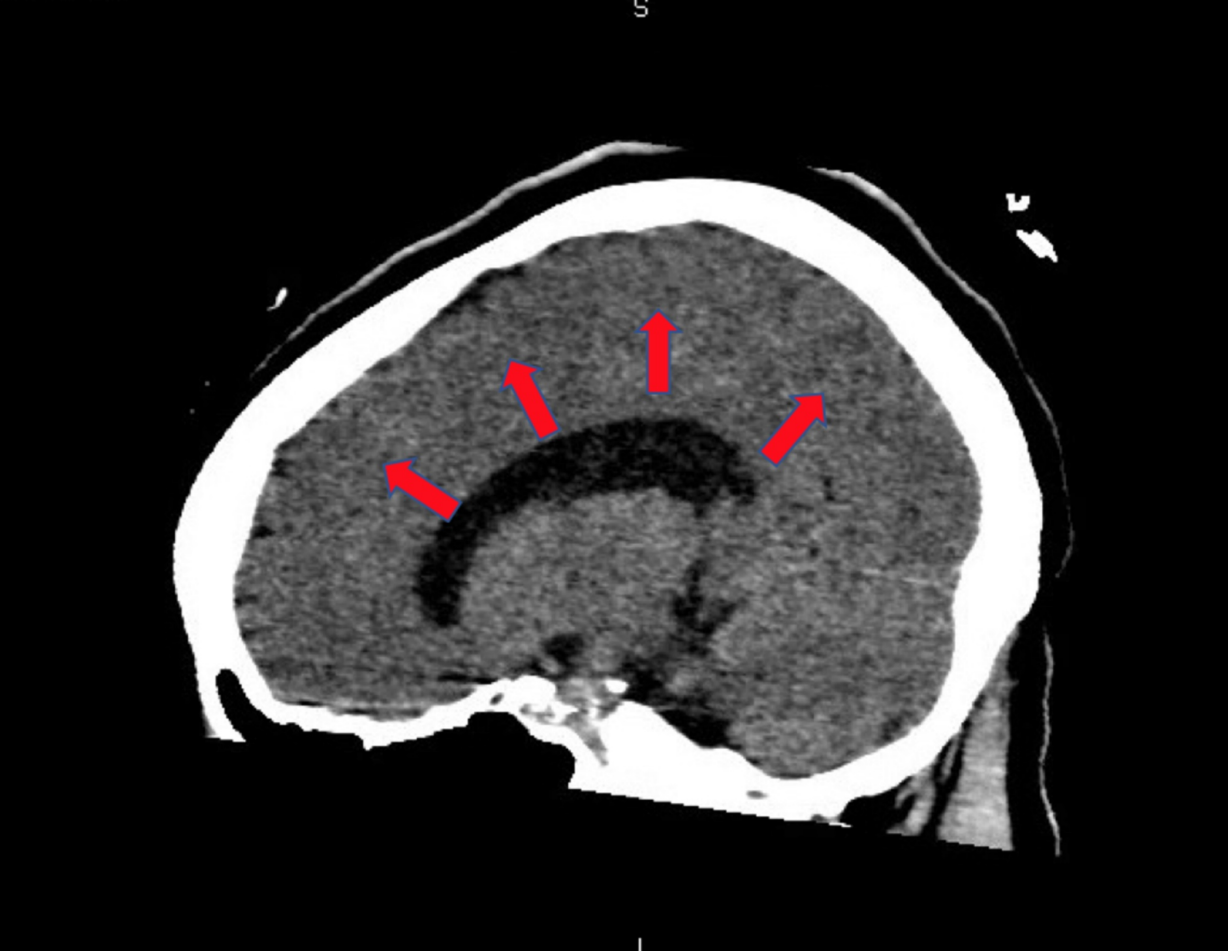
Computed tomography of the head without contrast shows loss of gray-white matter differentiation secondary to anoxic brain injury

The patient was transferred to the medical intensive care unit (ICU) intubated, sedated, and received multiple vasopressor infusions. Computed tomography angiogram (CTA) of the chest revealed extensive bilateral pulmonary emboli with findings suggestive of a right heart strain and dilatation of the main pulmonary artery reflective of pulmonary arterial hypertension, both confirming the diagnosis of massive pulmonary embolism (Figures 1-3). CT scan of the head showed a loss of gray-white matter differentiation and diffuse anoxic brain injury consistent with hypoxic-ischemic encephalopathy in the setting of cardiac arrest (Figures 4-5).

During her stay in the medical ICU, the patient showed consistent seizure-like activity, probably secondary to significant brain injury, and she was administered a propofol drip for epileptiform suppression. Two days later in the ICU, she became bradycardic (30 beats per minute) and ACLS was activated. Cardiopulmonary resuscitation was unsuccessful and the patient expired.

## Discussion

As previously discussed, ESRD is a major cause of morbidity and mortality in the US. In 2018, approximately 468,000 patients in the US were dependent on chronic hemodialysis, and of those, 51% relied on AVFs for hemodialysis access. The thrombophilic state in ESRD often leads to significant clotting in patients who rely on AVFs and grafts for hemodialysis access. This requires either surgical intervention to remove or thrombolyze the clots and to restore AVF function or create new access sites for hemodialysis.

In most cases of a malfunctioning AVF requiring thrombolysis, embolization is a relatively common but usually benign occurrence. Unless patients have a patent foramen ovale or some other cardiopulmonary defect, systemic embolization causing a serious cardiovascular accident is rare. The vast majority of clots that embolize the lungs are small enough to remain asymptomatic and they spontaneously resorb [5].

Nonetheless, this case report demonstrates that the embolization of certain clots, if large or resistant enough to heparinization and tPA, have the potential to cause cardiac arrest and death secondary to circulatory collapse. The risk is even greater in patients with other underlying comorbidities, such as this patient who had a history of severe SLE, which predisposes to hypercoagulability and local inflammation in various organs with resultant permanent organ damage. A literature review using the PubMed US Library of Medicine database over the past 20 years confirmed that only a handful of cases involving symptomatic PEs after AVF access declotting have been reported. In most of those cases, the circulatory collapse was temporary and patients were successfully discharged from the hospital following several days of critical care [6-8]. A death occurred in only one other case report in which the patient, like our patient, had significant preexisting cardiopulmonary morbidities that prevented successful resuscitation.

## Conclusions

It can be concluded from this case report that AVF access thrombectomy is not a benign, risk-free procedure. AVF thrombectomy can be complicated by lethal pulmonary embolisms, particularly in patients with other comorbid conditions predisposing to hypercoagulability or baseline cardiopulmonary dysfunction. Anesthesia providers should be prepared to manage massive pulmonary embolism in the case of AVF angiogram/angioplasty with anticoagulant treatment, such as low-molecular-weight heparin, factor Xa antagonists, and thrombolytic agents, such as tPA.

Although no such device currently exists, for high-risk cases, it may be worth considering the design and deployment of a filter in the proximal upper limb vasculature to prevent clots from reaching the pulmonary circulation, similar to the inferior vena cava filter deployed to catch deep vein thrombosis emboli from the lower extremities. Finally, in high-risk cases, it may be prudent to abandon the clotted access point altogether and either create a new AVF access point or consider alternative means of renal dialyses, such as peritoneal dialysis.
